# 10-[(4-Nitro­phen­yl)ethyn­yl]-10*H*-pheno­thia­zine

**DOI:** 10.1107/S2414314622009427

**Published:** 2022-10-27

**Authors:** Tsunehisa Okuno, Ikue Doi

**Affiliations:** a Department of Systems Engineering, Wakayama University, Sakaedani, Wakayama, 640-8510, Japan; Benemérita Universidad Autónoma de Puebla, México

**Keywords:** crystal structure, pheno­thia­zine, ynamine, heterocycle conformation.

## Abstract

The title compound, C_20_H_12_N_2_OS, is a 10-ethynyl-10*H*-pheno­thia­zine derivative. The pheno­thia­zine unit has a butterfly shape, where the folding angle between the two benzene rings is 153.87 (7)°, which is almost as in other reported pheno­thia­zine derivatives. The dihedral angle between the mean plane including the C atoms bonded to the pheno­thia­zine N atom and the benzene ring of the nitro­benzene group is 10.34 (5)°. The near planar geometry of the mol­ecule is reasonably explained by intra­molecular charge-transfer inter­actions.

## Abstract 2

In the title pheno­thia­zine derivative, the heterocycle displays a butterfly conformation.

## Graphical abstract

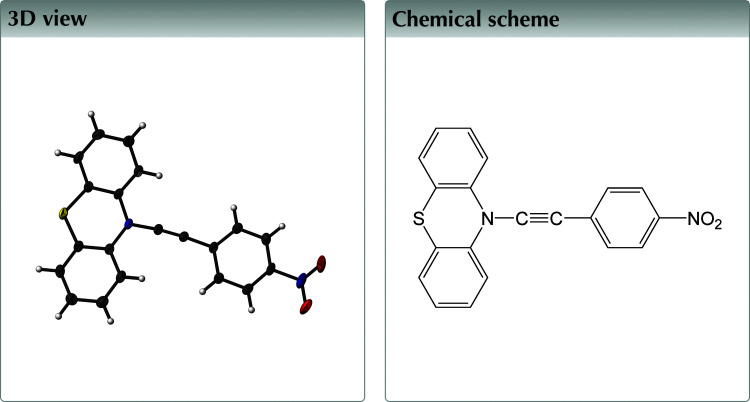

## Structure description

Pheno­thia­zines are known to be good electron donors and have attracted inter­est from the point of view of photo-induced electron transfer or magnetism (Sun *et al.*, 2004 ▸; Okamoto *et al.*, 2004 ▸; Okada *et al.*, 1996 ▸). A pheno­thia­zine derivative, 10-(prop-1-yn-1-yl)-10*H*-pheno­thia­zine, which incorporates an ynamine moiety, is well known as the first reported ynamine compound (Zaugg *et al.*, 1958 ▸), and its structure has already been studied (Umezono & Okuno, 2012 ▸). Other structures of some related derivatives have also been analysed (Umezono & Okuno, 2013 ▸; Umezono *et al.*, 2013 ▸).

**Figure 1:**
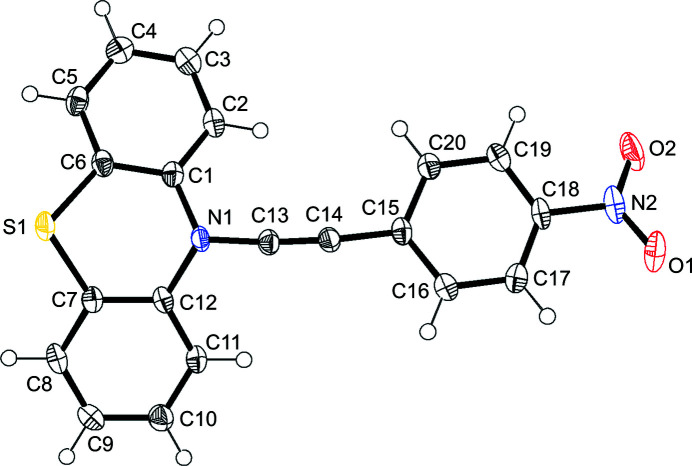
*ORTEP* view of the title compound with the atom-numbering scheme. Displacement ellipsoids are drawn at the 50% probability level and H atoms are shown as small spheres of arbitrary radii.

In the title compound, the pheno­thia­zine moiety has a butterfly structure, as shown in Fig. 1 ▸, in which the dihedral angle between the two benzene rings (the C1–C6 and C7–C12 mean planes) is 153.87 (7)°. The central six-membered ring has a boat conformation, in which the S1⋯N1 separation is 3.0565 (14) Å. The structure around the pheno­thia­zine nitro­gen atom is pyramidal, with atom N1 located 0.1271 (16) Å above the C1/C12/C13 plane. The dihedral angle between the C1/C12/C13 plane and the C15–C20 benzene ring is 10.34 (5)°. The mol­ecule is thus almost planar, and this feature is reasonably explained by intra­molecular charge-transfer inter­actions between pheno­thia­zine and nitro­phenyl units.

## Synthesis and crystallization

Single crystals suitable for X-ray analysis were obtained by concentration of a di­chloro­methane solution. The title compound was prepared through the Sonogashira-coupling reaction between 1-iodo-4-nitro­benzene and 10-ethynyl-10*H*-pheno­thia­zine, as follows: to a solution of 1-iodo-4-nitro­benzene (0.33 g, 1.3 mmol) and 10-ethynyl-10*H*-pheno­thia­zine (0.30 g, 1.3 mmol) in 13 ml of THF and tri­ethyl­amine (1:1 *v*/*v*), tetra­kis­(tri­phenyl­phosphine)palladium(0) (0.093 g, 0.080 mmol) and copper(I) iodide (8.0 mg, 0.040 mmol) were added. The solution was stirred for 20 h and filtrated. The filtrate was concentrated and the residue was extracted with CHCl_3_. The organic layer was washed with water, dried over Na_2_SO_4_ and concentrated under reduced pressure. The residue was purified by gel permeation chromatography to give 32 mg (6.9% yield) of the title compound, as pale-red crystals. ^1^H NMR (CDCl_3_): δ = 8.21 (*d*, *J* = 9.0 Hz, 0.8 Hz, 2H), 7.58 (*d*, *J* = 9.0 Hz, 2H), 7.48 (*d*, *J* = 7.3 Hz, 2H), 7.26 (*t*, *J* = 7.3 Hz, 2H), 7.17 (*d*, *J* = 6.5 Hz, 2H), 7.10 (*t*, *J* = 6.5 Hz, 2H).

## Refinement

Crystal data, data collection and structure refinement details are summarized in Table 1 ▸.

## Supplementary Material

Crystal structure: contains datablock(s) I. DOI: 10.1107/S2414314622009427/bh4070sup1.cif

Structure factors: contains datablock(s) I. DOI: 10.1107/S2414314622009427/bh4070Isup2.hkl

Supporting information file. DOI: 10.1107/S2414314622009427/bh4070Isup3.cml

CCDC reference: 2209381

Additional supporting information:  crystallographic information; 3D view; checkCIF report

## Figures and Tables

**Table 1:** Experimental details

Crystal data	
Chemical formula	C_20_H_12_N_2_O_2_S
*M*_r_	344.39
Crystal system, space group	Triclinic, *P* 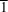
Temperature (K)	93
*a*, *b*, *c* (Å)	8.1891 (15), 8.2417 (15), 12.813 (3)
α, β, γ (°)	81.632 (9), 81.394 (10), 66.649 (8)
*V* (Å^3^)	781.4 (3)
*Z*	2
Radiation type	Mo *K*α
μ (mm^−1^)	0.22
Crystal size (mm)	0.15 × 0.12 × 0.05

Data collection	
Diffractometer	Rigaku Saturn724+
Absorption correction	Numerical (*NUMABS*; Rigaku, 1999 ▸)
*T*_min_, *T*_max_	0.977, 0.988
No. of measured, independent and observed [*F* ^2^ > 2.0σ(*F* ^2^)] reflections	5406, 2701, 2234
*R*_int_	0.021
(sin θ/λ)_max_ (Å^−1^)	0.595

Refinement	
*R*[*F* ^2^ > 2σ(*F* ^2^)], *wR*(*F* ^2^), *S*	0.037, 0.100, 1.09
No. of reflections	2701
No. of parameters	226
H-atom treatment	H-atom parameters constrained
Δρ_max_, Δρ_min_ (e Å^−3^)	0.24, −0.22

Computer programs: *CrystalClear* (Rigaku, 2008 ▸), *SHELXD2013/2* (Sheldrick, 2008 ▸), *SHELXL2014/7* (Sheldrick, 2015 ▸), *ORTEP-3 for Windows* (Farrugia, 2012 ▸) and *CrystalStructure* (Rigaku, 2019 ▸).

## full crystallographic data

### Crystal data

**Table:**

C_20_H_12_N_2_O_2_S	*Z* = 2
*M**_r_* = 344.39	*F*(000) = 356.00
Triclinic, *P*1	*D*_x_ = 1.464 Mg m^−^^3^
*a* = 8.1891 (15) Å	Mo *K*α radiation, λ = 0.71075 Å
*b* = 8.2417 (15) Å	Cell parameters from 2498 reflections
*c* = 12.813 (3) Å	θ = 1.6–31.3°
α = 81.632 (9)°	µ = 0.22 mm^−^^1^
β = 81.394 (10)°	*T* = 93 K
γ = 66.649 (8)°	Block, red
*V* = 781.4 (3) Å^3^	0.15 × 0.12 × 0.05 mm

### Data collection

**Table:**

Rigaku Saturn724+ diffractometer	2234 reflections with *F*^2^ > 2.0σ(*F*^2^)
Detector resolution: 7.111 pixels mm^-1^	*R*_int_ = 0.021
ω scans	θ_max_ = 25.0°, θ_min_ = 1.6°
Absorption correction: numerical (NUMABS; Rigaku, 1999)	*h* = −9→9
*T*_min_ = 0.977, *T*_max_ = 0.988	*k* = −9→9
5406 measured reflections	*l* = −11→15
2701 independent reflections	

### Refinement

**Table:**

Refinement on *F*^2^	Secondary atom site location: difference Fourier map
*R*[*F*^2^ > 2σ(*F*^2^)] = 0.037	Hydrogen site location: inferred from neighbouring sites
*wR*(*F*^2^) = 0.100	H-atom parameters constrained
*S* = 1.09	*w* = 1/[σ^2^(*F*_o_^2^) + (0.0537*P*)^2^ + 0.2098*P*] where *P* = (*F*_o_^2^ + 2*F*_c_^2^)/3
2701 reflections	(Δ/σ)_max_ = 0.001
226 parameters	Δρ_max_ = 0.24 e Å^−^^3^
0 restraints	Δρ_min_ = −0.22 e Å^−^^3^
Primary atom site location: structure-invariant direct methods	

### Special details

**Table:**

Refinement. The C-bound H atoms were placed in ideal positions and were refined as riding on their parent C atoms. *U*_iso_(H) values of the H atoms were set at 1.2*U*_eq_(parent atom).

### Fractional atomic coordinates and isotropic or equivalent isotropic displacement parameters (Å^2^)

**Table:**

	*x*	*y*	*z*	*U*_iso_*/*U*_eq_	
S1	−0.18967 (6)	1.42062 (6)	0.10364 (4)	0.02912 (16)	
O1	0.67949 (19)	−0.06179 (18)	0.41540 (13)	0.0439 (4)	
O2	0.5066 (2)	−0.10830 (18)	0.32062 (13)	0.0465 (4)	
N1	−0.02003 (19)	1.04512 (18)	0.21162 (11)	0.0224 (3)	
N2	0.5590 (2)	−0.0089 (2)	0.35670 (14)	0.0341 (4)	
C1	−0.1331 (2)	1.0636 (2)	0.13171 (13)	0.0211 (4)	
C2	−0.1530 (2)	0.9172 (2)	0.10350 (14)	0.0256 (4)	
H2	−0.0962	0.8040	0.1403	0.031*	
C3	−0.2544 (2)	0.9333 (3)	0.02236 (15)	0.0283 (4)	
H3	−0.2634	0.8309	0.0022	0.034*	
C4	−0.3429 (2)	1.0989 (2)	−0.02954 (15)	0.0279 (4)	
H4	−0.4134	1.1107	−0.0849	0.034*	
C5	−0.3276 (2)	1.2470 (2)	0.00017 (14)	0.0262 (4)	
H5	−0.3903	1.3608	−0.0341	0.031*	
C6	−0.2220 (2)	1.2315 (2)	0.07908 (14)	0.0231 (4)	
C7	−0.1384 (2)	1.3617 (2)	0.23645 (14)	0.0224 (4)	
C8	−0.1760 (2)	1.4970 (2)	0.30086 (15)	0.0263 (4)	
H8	−0.2378	1.6169	0.2748	0.032*	
C9	−0.1241 (2)	1.4584 (2)	0.40224 (15)	0.0274 (4)	
H9	−0.1490	1.5515	0.4454	0.033*	
C10	−0.0355 (2)	1.2829 (2)	0.44086 (15)	0.0283 (4)	
H10	0.0026	1.2558	0.5100	0.034*	
C11	−0.0028 (2)	1.1470 (2)	0.37808 (14)	0.0244 (4)	
H11	0.0552	1.0270	0.4053	0.029*	
C12	−0.0541 (2)	1.1850 (2)	0.27614 (14)	0.0216 (4)	
C13	0.0882 (2)	0.8781 (2)	0.24405 (13)	0.0226 (4)	
C14	0.1838 (2)	0.7265 (2)	0.26460 (14)	0.0234 (4)	
C15	0.2884 (2)	0.5416 (2)	0.28480 (13)	0.0217 (4)	
C16	0.4081 (2)	0.4783 (2)	0.36260 (14)	0.0255 (4)	
H16	0.4267	0.5599	0.4001	0.031*	
C17	0.4991 (2)	0.2982 (2)	0.38510 (15)	0.0268 (4)	
H17	0.5802	0.2548	0.4379	0.032*	
C18	0.4703 (2)	0.1825 (2)	0.32941 (15)	0.0251 (4)	
C19	0.3582 (2)	0.2398 (2)	0.24936 (15)	0.0255 (4)	
H19	0.3436	0.1570	0.2110	0.031*	
C20	0.2681 (2)	0.4202 (2)	0.22653 (14)	0.0244 (4)	
H20	0.1919	0.4623	0.1711	0.029*	

### Atomic displacement parameters (Å^2^)

**Table:**

	*U*^11^	*U*^22^	*U*^33^	*U*^12^	*U*^13^	*U*^23^
S1	0.0395 (3)	0.0158 (2)	0.0288 (3)	−0.0084 (2)	−0.0061 (2)	0.00428 (18)
O1	0.0330 (8)	0.0244 (7)	0.0613 (10)	0.0006 (6)	−0.0119 (7)	0.0094 (7)
O2	0.0478 (9)	0.0152 (7)	0.0734 (12)	−0.0077 (6)	−0.0084 (8)	−0.0054 (7)
N1	0.0264 (8)	0.0130 (7)	0.0236 (8)	−0.0034 (6)	−0.0026 (6)	−0.0003 (6)
N2	0.0272 (9)	0.0167 (8)	0.0481 (11)	−0.0020 (7)	0.0046 (8)	0.0011 (8)
C1	0.0190 (8)	0.0194 (9)	0.0211 (9)	−0.0052 (7)	0.0021 (7)	−0.0009 (7)
C2	0.0245 (9)	0.0182 (9)	0.0299 (10)	−0.0057 (7)	0.0014 (8)	−0.0004 (7)
C3	0.0262 (9)	0.0263 (10)	0.0327 (10)	−0.0113 (8)	0.0024 (8)	−0.0056 (8)
C4	0.0221 (9)	0.0333 (10)	0.0272 (10)	−0.0098 (8)	−0.0005 (7)	−0.0033 (8)
C5	0.0213 (9)	0.0238 (9)	0.0260 (10)	−0.0033 (8)	0.0007 (7)	0.0026 (7)
C6	0.0235 (9)	0.0187 (9)	0.0228 (9)	−0.0055 (7)	0.0023 (7)	−0.0012 (7)
C7	0.0221 (9)	0.0183 (9)	0.0251 (9)	−0.0074 (7)	−0.0011 (7)	0.0007 (7)
C8	0.0253 (9)	0.0152 (9)	0.0356 (11)	−0.0067 (7)	0.0013 (8)	−0.0013 (7)
C9	0.0298 (10)	0.0238 (10)	0.0314 (10)	−0.0131 (8)	0.0025 (8)	−0.0083 (8)
C10	0.0328 (10)	0.0252 (10)	0.0274 (10)	−0.0122 (8)	−0.0028 (8)	−0.0012 (8)
C11	0.0261 (9)	0.0174 (9)	0.0268 (10)	−0.0068 (7)	−0.0019 (7)	0.0016 (7)
C12	0.0207 (8)	0.0155 (8)	0.0259 (9)	−0.0054 (7)	0.0016 (7)	−0.0024 (7)
C13	0.0257 (9)	0.0181 (9)	0.0212 (9)	−0.0068 (8)	−0.0008 (7)	0.0002 (7)
C14	0.0270 (9)	0.0183 (9)	0.0228 (9)	−0.0067 (8)	−0.0022 (7)	−0.0018 (7)
C15	0.0213 (9)	0.0166 (8)	0.0230 (9)	−0.0052 (7)	0.0025 (7)	0.0004 (7)
C16	0.0257 (9)	0.0203 (9)	0.0290 (10)	−0.0074 (8)	−0.0005 (8)	−0.0036 (7)
C17	0.0222 (9)	0.0230 (9)	0.0302 (10)	−0.0045 (8)	−0.0034 (8)	0.0016 (8)
C18	0.0205 (9)	0.0142 (9)	0.0338 (10)	−0.0021 (7)	0.0026 (8)	−0.0003 (7)
C19	0.0245 (9)	0.0194 (9)	0.0309 (10)	−0.0073 (8)	0.0030 (8)	−0.0060 (7)
C20	0.0241 (9)	0.0209 (9)	0.0256 (9)	−0.0066 (7)	−0.0007 (7)	−0.0020 (7)

### Geometric parameters (Å, º)

**Table:**

S1—C6	1.7591 (19)	C8—C9	1.381 (3)
S1—C7	1.7661 (19)	C8—H8	0.9500
O1—N2	1.231 (2)	C9—C10	1.390 (3)
O2—N2	1.232 (2)	C9—H9	0.9500
N1—C13	1.353 (2)	C10—C11	1.390 (3)
N1—C12	1.430 (2)	C10—H10	0.9500
N1—C1	1.434 (2)	C11—C12	1.386 (3)
N2—C18	1.464 (2)	C11—H11	0.9500
C1—C2	1.383 (3)	C13—C14	1.198 (2)
C1—C6	1.406 (2)	C14—C15	1.428 (2)
C2—C3	1.385 (3)	C15—C16	1.401 (3)
C2—H2	0.9500	C15—C20	1.405 (2)
C3—C4	1.388 (3)	C16—C17	1.380 (2)
C3—H3	0.9500	C16—H16	0.9500
C4—C5	1.386 (3)	C17—C18	1.379 (3)
C4—H4	0.9500	C17—H17	0.9500
C5—C6	1.386 (3)	C18—C19	1.384 (3)
C5—H5	0.9500	C19—C20	1.382 (2)
C7—C8	1.392 (2)	C19—H19	0.9500
C7—C12	1.396 (2)	C20—H20	0.9500
			
C6—S1—C7	100.18 (8)	C8—C9—H9	120.1
C13—N1—C12	118.98 (15)	C10—C9—H9	120.1
C13—N1—C1	116.86 (15)	C9—C10—C11	119.86 (17)
C12—N1—C1	121.74 (13)	C9—C10—H10	120.1
O1—N2—O2	123.51 (16)	C11—C10—H10	120.1
O1—N2—C18	118.62 (17)	C12—C11—C10	120.61 (16)
O2—N2—C18	117.86 (17)	C12—C11—H11	119.7
C2—C1—C6	119.15 (17)	C10—C11—H11	119.7
C2—C1—N1	120.85 (15)	C11—C12—C7	119.39 (16)
C6—C1—N1	119.98 (16)	C11—C12—N1	120.54 (15)
C1—C2—C3	120.95 (17)	C7—C12—N1	120.07 (16)
C1—C2—H2	119.5	C14—C13—N1	174.55 (19)
C3—C2—H2	119.5	C13—C14—C15	175.25 (19)
C2—C3—C4	120.04 (18)	C16—C15—C20	119.27 (15)
C2—C3—H3	120.0	C16—C15—C14	121.62 (16)
C4—C3—H3	120.0	C20—C15—C14	119.10 (16)
C5—C4—C3	119.34 (18)	C17—C16—C15	120.40 (17)
C5—C4—H4	120.3	C17—C16—H16	119.8
C3—C4—H4	120.3	C15—C16—H16	119.8
C4—C5—C6	121.03 (16)	C18—C17—C16	118.75 (17)
C4—C5—H5	119.5	C18—C17—H17	120.6
C6—C5—H5	119.5	C16—C17—H17	120.6
C5—C6—C1	119.45 (17)	C17—C18—C19	122.61 (16)
C5—C6—S1	118.75 (13)	C17—C18—N2	119.09 (17)
C1—C6—S1	121.63 (14)	C19—C18—N2	118.29 (17)
C8—C7—C12	119.73 (17)	C20—C19—C18	118.49 (17)
C8—C7—S1	118.41 (13)	C20—C19—H19	120.8
C12—C7—S1	121.78 (14)	C18—C19—H19	120.8
C9—C8—C7	120.60 (16)	C19—C20—C15	120.37 (17)
C9—C8—H8	119.7	C19—C20—H20	119.8
C7—C8—H8	119.7	C15—C20—H20	119.8
C8—C9—C10	119.75 (17)		
			
C13—N1—C1—C2	11.1 (2)	C10—C11—C12—C7	0.3 (3)
C12—N1—C1—C2	−151.03 (16)	C10—C11—C12—N1	179.89 (16)
C13—N1—C1—C6	−167.32 (15)	C8—C7—C12—C11	−2.3 (3)
C12—N1—C1—C6	30.6 (2)	S1—C7—C12—C11	174.37 (13)
C6—C1—C2—C3	2.0 (3)	C8—C7—C12—N1	178.07 (16)
N1—C1—C2—C3	−176.47 (15)	S1—C7—C12—N1	−5.2 (2)
C1—C2—C3—C4	−2.2 (3)	C13—N1—C12—C11	−10.9 (2)
C2—C3—C4—C5	0.5 (3)	C1—N1—C12—C11	150.89 (16)
C3—C4—C5—C6	1.5 (3)	C13—N1—C12—C7	168.71 (15)
C4—C5—C6—C1	−1.7 (3)	C1—N1—C12—C7	−29.5 (2)
C4—C5—C6—S1	173.52 (13)	C20—C15—C16—C17	−2.9 (3)
C2—C1—C6—C5	0.0 (2)	C14—C15—C16—C17	175.91 (16)
N1—C1—C6—C5	178.45 (15)	C15—C16—C17—C18	0.2 (3)
C2—C1—C6—S1	−175.11 (13)	C16—C17—C18—C19	2.3 (3)
N1—C1—C6—S1	3.3 (2)	C16—C17—C18—N2	−176.74 (15)
C7—S1—C6—C5	155.97 (14)	O1—N2—C18—C17	−12.3 (2)
C7—S1—C6—C1	−28.87 (16)	O2—N2—C18—C17	166.68 (17)
C6—S1—C7—C8	−153.28 (14)	O1—N2—C18—C19	168.69 (17)
C6—S1—C7—C12	29.96 (16)	O2—N2—C18—C19	−12.4 (2)
C12—C7—C8—C9	2.6 (3)	C17—C18—C19—C20	−1.8 (3)
S1—C7—C8—C9	−174.25 (14)	N2—C18—C19—C20	177.16 (15)
C7—C8—C9—C10	−0.7 (3)	C18—C19—C20—C15	−1.0 (2)
C8—C9—C10—C11	−1.3 (3)	C16—C15—C20—C19	3.3 (3)
C9—C10—C11—C12	1.5 (3)	C14—C15—C20—C19	−175.53 (16)
